# High‐Definition Transcranial Direct Current Stimulation Enhances Exercise‐Induced Hypoalgesia in Healthy Individuals: An fNIRS Study

**DOI:** 10.1002/brb3.70595

**Published:** 2025-06-10

**Authors:** Ruihan Wan, Haozhi Zhao, Xue Jiang, Beibei Feng, Yafei Wang, Chen Gong, Yangyang Lin, Wangwang Yan, Yixuan Ku, Yuling Wang

**Affiliations:** ^1^ Department of Rehabilitation Medicine The Sixth Affiliated Hospital of Sun Yat‐Sen University Guangzhou Guangdong China; ^2^ College of Rehabilitation Medicine Fujian University of Traditional Chinese Medicine Fuzhou Fujian China; ^3^ Guangdong Provincial Clinical Research Center for Rehabilitation Medicine Guangzhou Guangdong China; ^4^ Biomedical Innovation Center The Sixth Affiliated Hospital of Sun Yat‐Sen University Guangzhou Guangdong China; ^5^ College of Exercise and Health Shenyang Sport University Shenyang Liaoning China; ^6^ Department of Sport Rehabilitation Shanghai University of Sport Yangpu Shanghai China; ^7^ Brain and Mental Well‐Being Center, Department of Psychology Sun Yat‐Sen University Guangzhou Guangdong China; ^8^ Peng Cheng Laboratory Shenzhen Guangdong China

**Keywords:** exercise‐induced hypoalgesia, fNIRS, HD‐tDCS, healthy adults

## Abstract

**Background:**

This study investigates whether anodal high‐definition transcranial direct current stimulation (HD‐tDCS) enhances exercise‐induced hypoalgesia (EIH) and explores brain plasticity changes using functional near‐infrared spectroscopy (fNIRS).

**Methods:**

Thirty‐nine participants were randomly assigned to either the active (*n* = 19) or sham HD‐tDCS (*n* = 20) group. Both groups performed 25 min of moderate‐intensity aerobic exercise followed by 20 min of either active or sham HD‐tDCS applied to the left primary motor cortex (M1). Primary outcome: pressure pain threshold (PPT) at a local site. Secondary outcomes: PPT at a remote site, cold pain threshold (CPT), and brain activation changes via fNIRS during the cold pressor test.

**Results:**

Both groups showed significant increases in PPT_leg_ (active: from 42.21 ± 11.77 N to 51.29 ± 12.75 N; sham: from 41.41 ± 9.73 N to 45.29 ± 12.05 N, *p* < 0.001) and PPT_forearm_ (active: from 31.69 ± 6.06 N to 36.99 ± 6.35 N; sham: from 32.66 ± 7.34 N to 37.08 ± 10.56 N, *p* < 0.001). The active group showed a significantly greater increase in PPT_leg_ compared to the sham group (9.08 ± 8.01 N vs. 3.69 ± 4.36 N, *p* < 0.012). fNIRS analysis revealed significant changes in specific cortical channels in the active group (*p* < 0.05), with a negative correlation between cortical activation in CH16 and PPTleg (*r* = −0.405, *p* = 0.011).

**Conclusion:**

HD‐tDCS over M1 enhances EIH and is associated with increased brain activation in sensory‐motor processing areas.

**Trial Registration:**

Clinical trial registration: ChiCTR2100048146

## Graphical abstract

Anodal HD‐tDCS over motor cortex enhances exercise‐induced hypoalgesia in healthy adults. functional near‐infrared spectroscopy reveals reduced pain‐processing cortical activation. These findings in healthy individuals may inform future investigations into enhancing exercise adherence strategies for pain populations.

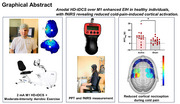

## 1 Introduction

With its alarmingly high prevalence, chronic musculoskeletal pain has become a debilitating problem that raises global public concern, which has resulted in burdensome medical and socioeconomic costs (Lam et al. 2024; Mills et al. 2019). As estimated, more than 320 million adults are affected by musculoskeletal pain every year globally, and the global cost of managing pain is estimated at $560 billion annually. A large majority of those who are suffering from chronic musculoskeletal pain also experience severe psychological distress (Spoonemore et al. 2023; Zelaya et al. 2020).

Extensive evidence demonstrates the benefit of regular exercise for pain alleviation (Gilanyi et al. 2023; Wewege and Jones 2021; Zheng et al. 2021). Exercise‐induced hypoalgesia (EIH, the phenomenon of reduced pain sensitivity following physical activity) refers to the presentation of lessened pain sensitivity or diminished pain perception after physical exercise (Wewege and Jones 2021; Rice et al. 2019). EIH occurs in both healthy individuals and those with chronic pain, and the response of hypoalgesia is thought to be an indicator associated with the effectiveness of pain treatments (Zheng et al. 2021; Gomolka et al. 2019). Vaegter et al. (2017) found that preoperative EIH was positively correlated with pain reduction after total knee replacement, suggesting that EIH could be used as a predictor for responsiveness to pain‐relieving interventions. It is believed that the transient alleviation in pain perception after exercise not only relieves their pain experience, but also increases their confidence to maintain physical activity (Rice et al. 2019). However, the EIH effect merely lasts 30 min (Senarath et al. 2023), and it is generally impaired among individuals with chronic pain or persistent pain (Song et al. 2022). These limitations highlight the need for interventions that can amplify or prolong EIH responses. In this context, high‐definition transcranial direct current stimulation (HD‐tDCS) emerges as a promising candidate.

HD‐tDCS, as one of the more promising noninvasive neuromodulation techniques (Kuo et al. 2013), may address EIH's transient nature by “priming” neural circuits involved in pain processing. Our previous findings have verified that anodal HD‐tDCS over the left M1 relieved experimentally‐induced pain compared to sham HD‐tDCS in healthy adults (Wan et al. 2021; Jiang et al. 2022). Specifically, its ability to enhance cortical excitability through targeted anodal stimulation could facilitate endogenous pain inhibitory systems. This is supported by evidence that enhanced motor corticospinal excitability correlates with strengthened antinociceptive mechanisms (Granovsky et al. 2019; Zappasodi et al. 2019). Compared with traditional tDCS, HD‐tDCS induces prolonged neuromodulatory after‐effects lasting over 2 h (Kuo et al. 2013), which may underlie its enhanced analgesic potential, thus suggesting its utility in overcoming the short‐lived efficacy of EIH. Furthermore, HD‐tDCS pretreatment has been shown to enhance subsequent treatment outcomes by modulating neural plasticity (Chang et al. 2017; Pilloni et al. 2020), providing a mechanistic rationale for combining it with EIH protocols.

To investigate the neural correlates of HD‐tDCS‐enhanced EIH, we employed functional near‐infrared spectroscopy (fNIRS), a noninvasive neuroimaging technique that quantifies cortical hemodynamic changes via near‐infrared light. Unlike fMRI, fNIRS offers high tolerance to motion artifacts and real‐time monitoring of brain activity during dynamic tasks (for e.g., pain stimulation) (Sorkpor et al. 2023; Karunakaran et al. 2021), making it uniquely suited to capture exercise‐induced cortical plasticity in sensory‐motor regions. Specifically, fNIRS allows simultaneous measurement of prefrontal and motor cortex activation during the cold pressor test, enabling us to link HD‐tDCS‐induced neuromodulation with EIH. To our knowledge, this is the first study integrating HD‐tDCS and fNIRS to decipher EIH mechanisms, this is the first study to combine HD‐tDCS and fNIRS for investigating EIH mechanisms. We hypothesized that, compared with the sham HD‐tDCS group, the active HD‐tDCS group would be associated with greater EIH responses among individuals with experimental pain, potentially through altered brain activation in sensory‐motor processing areas.

## 2 Methods

### 2.1 Study Design

This was a single‐center, randomized, single‐blinded (participants), parallel controlled trial. The simple randomization method was applied for randomization in this study. A statistician who is not involved in this study used SPSS 24.0 (IBM, Inc., Chicago, USA) software to generate a random number sequence and randomly assign the participants into two groups namely the active HD‐tDCS group and the sham HD‐tDCS group via opaque sealed envelopes. Each participant was blind to group allocation. Intervention administrators were not blinded to group allocation, outcome assessors and data analysts maintained complete blinding throughout the study period.

The study was performed in accordance with the Declaration of Helsinki. It was approved by the Ethics Committee of the Sixth Affiliated Hospital of Sun Yat‐Sen University, Guangzhou, China (2021ZSLYEC‐350) and registered at the Chinese Clinical Trial Registry (http://www.chictr.org.cn; ChiCTR2100048146).

### 2.2 Participants

Based on the previously published study by Borovskis et al. (2021), the effect size (Cohen's *d*) of active versus sham tDCS on postexercise local PPT was calculated to be 1.11. Given the similarity in intervention protocol and outcome measures, this value was adopted as the estimated effect size for sample size calculation in the present study. Using G*Power 3.1.9.7, a two‐tailed independent samples *t*‐test with an alpha level of 0.05 and power (1 − β) of 0.80 indicated that a total sample size of 28 participants (14 per group) would be required to detect a significant between‐group difference. To account for an anticipated dropout rate of 20%, the final target sample size was adjusted to 35 participants (28 / [1 − 0.20] ≈ 35). From April to October 2022, a total of 43 eligible participants were enrolled in the study, ensuring sufficient statistical power for hypothesis testing. The screenshot of the power analysis output has been provided in File S1.

The inclusion criteria were as follows: right‐handed (Oldfield 1971) and right‐leg dominant, defined by standard physical assessment in relevant literature (Gomolka et al. 2019); 18–40 years old; engaging in less than 10 h of organized sports per week; no habitual smoking or alcohol drinking; not in menstrual cycle or pregnancy; no acute or persistent pain requiring medical attention or analgesic use within 3 months; no severe psychiatric and mental illnesses or any drug abuse within 6 months; no current hand injuries or open wounds; and no contraindications for tDCS (for e.g., implantable medical devices or history of epilepsy). The withdrawal criteria were the occurrence of severe adverse events (for e.g., nausea, vomiting, palpitations, etc.) during the study or participants' voluntary withdrawal from the trial for any reason at any time.

All participants were required to sign an informed consent before participation in the study.

### 2.3 Experimental Procedures

**FIGURE 1:**
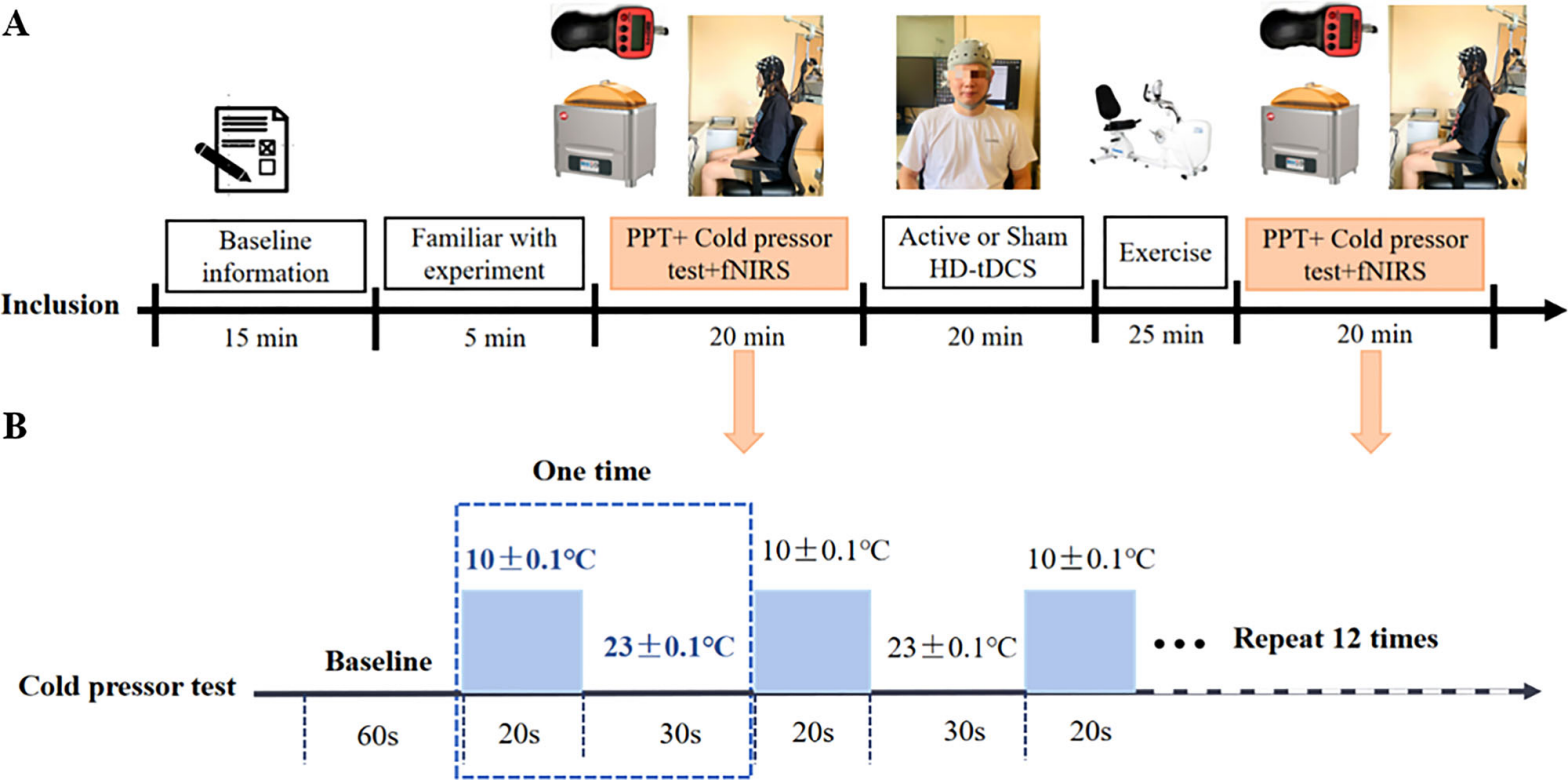
Schematic illustration of the experimental procedures. fNIRS: functional near‐infrared spectroscopy; PPT: pressure pain threshold.

The study was conducted in an undisturbed laboratory room at a temperature of 23°C. Participants were instructed to avoid vigorous physical activity, caffeine, and tobacco for 24 h before the experiment. As shown in Figure 1A, first, the baseline information of all eligible participants was collected, including Beck depression inventory‐II (BDI‐II), pain catastrophizing scale (PCS), state‐trait anxiety inventory (STAI), fear of pain questionnaire (FPQ), and international physical activity questionnaire (IPAQ). Lower limb dominance was assessed via a standardized bilateral forward pushing test: participants stood upright while an experimenter applied gentle forward pressure to both shoulders; the leg used to regain balance (i.e., the first leg stepping forward) was recorded as the dominant limb (Gomolka et al. 2019). Following protocol familiarization (verbal and visual instructions on procedures such as PPT, CPT, and tolerance thresholds without actual pain stimulation), baseline pain sensitivity measurements were conducted immediately prior to the intervention to prevent preconditioning effects.

Then the brain stimulation session started. In the active HD‐tDCS group, 2 mA electrical current was applied on the left M1 for 20 min, including 30 s ramp up (from 0 to 2 mA) at the beginning of stimulation and 30 s ramp down (from 2 to 0 mA) at the end of stimulation. In the sham group, the current was applied for the first and last 30 s to mimic active stimulation but without delivering therapeutic effects.

Subsequently, participants were requested to perform 25‐min moderate‐intensity aerobic exercise, of which the intensity was set at 64%–76% of the individual's maximum heart rate (HR; HRmax = 220 − age). A Polar HR monitor was used to collect HR during the whole process of aerobic exercise. Meanwhile, the rating of perceived exertion (RPE) was recorded every 5 min, and the physical activity enjoyment scale (PACES) was collected after the exercise.

Primary (the changes of PPT at the local site) and secondary outcomes (the changes of PPT at the remote site, CPT and tolerance, and hemodynamic changes in the frontal and bilateral motor cortex) were collected before and immediately after the intervention.

### 2.4 Intervention

#### 2.4.1 HD‐tDCS

**FIGURE 2:**
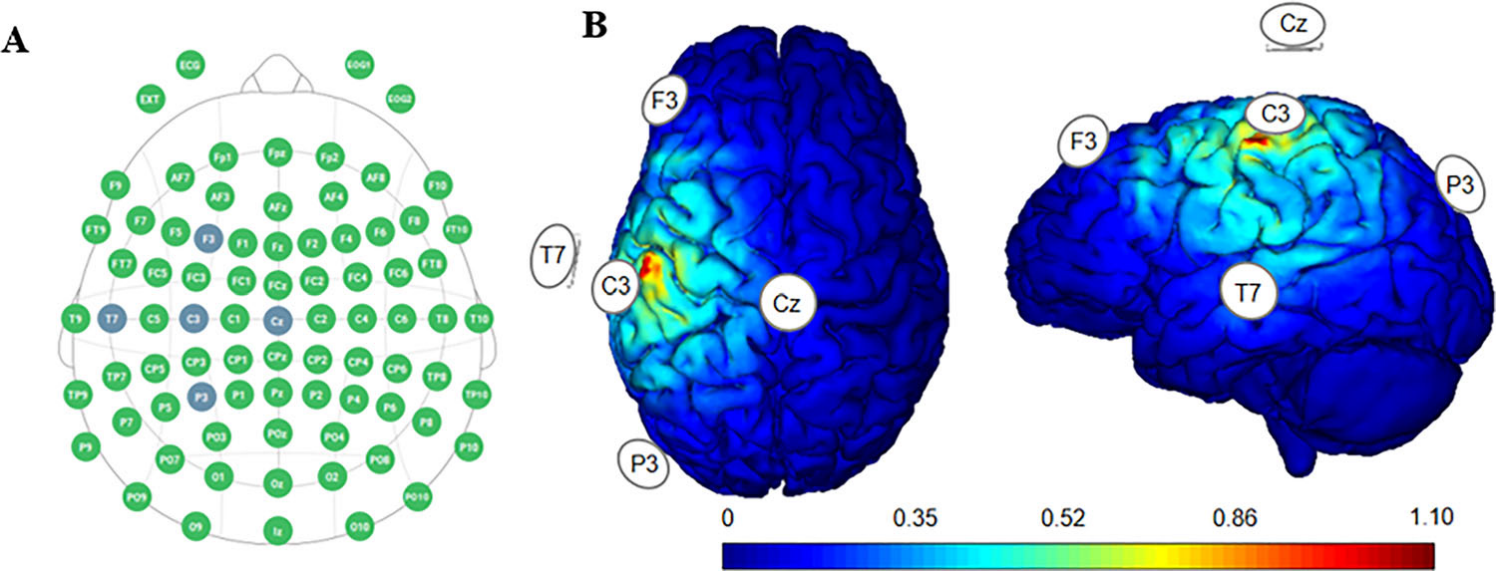
The stimulation paradigm of HD‐tDCS. (A) illustrates the electrode placement based on the international 10‐10 EEG system; (B) shows the electric field distribution (superior view and lateral view) simulated via neurostimulation software. All values (V/m) are positive, representing the magnitude of the electric field intensity.

HD‐tDCS was administered by an eight‐channel noninvasive electrical stimulator (Starstim 8, Neuroelectrics, Spain) with Ag/AgCl gelled electrodes placed on the left M1 (2 mA of a central electrode: C3; 500 µA of four return electrodes: Cz, F3, T7, and P3) to arrange in a 4 × 1 ring configuration (Figure 2). The electrode position was identified in accordance with the standard International 10–10 EEG System (Jurcak et al. 2007) and previous studies (Wan et al. 2021; Flood et al. 2016, 2017). The participants' head circumferences were measured to select correctly sized EEG caps (NE019, Neuroelectrics, Spain) before the stimulation, and the symmetry of Cz, nasion, and the preauricular points anterior to the left and right ears was checked to identify the accuracy of stimulation location. The application of HD‐tDCS strictly adhered to the established protocol in previous literature (Villamar et al. 2013; Hampstead et al. 2020).

In the active stimulation group, the current was applied over the left M1 at the intensity of 2 mA for 20 min, including a 30 s ramp up at the beginning of stimulation and a 30 s ramp down at the end of stimulation. For the sham stimulation, the current was delivered only during the first and last 30 s. During the whole process of stimulation, the impedance values were recorded automatically and maintained at a safety range of below 10 kΩ.

After the tDCS intervention, blinding effectiveness was assessed in the two groups. Participants were also asked to complete a standard adverse effect questionnaire. The skin condition underneath the stimulation electrode was carefully examined and recorded.

#### 2.4.2 Moderate Intensity Aerobic Exercise

The exercise paradigm was performed on an HR‐controlled cycle ergometer (Monark, Vansbro, Sweden) following active or sham HD‐tDCS. Participants were instructed to sit in a pleasant posture on the ergometer. An HR belt (Polar Electro H10, Malaysia) was worn to monitor and ensure the stability of HR during the whole cycling exercise. All participants were required to cycle for 25 min, including a 5 min warm‐up period (initial 2‐min cycling workload of 30 Watts, gradually accelerating in the next 3 min until the participants reached their target HR range); 15 min of exercise while remaining at their target HR range by adjusting the rotational speed and resistance (at the same time the operator provided verbal encouragement); and a 5 min recovery period (cool down at 30 Watts) (Mellow et al. 2020; Xu et al. 2019, 2020). The target HR was calculated in accordance with the American College of Sports Medicine guideline for healthy adults with the following formula: Target HR range = ([220 − age] × [64%–76%] intensity); HRmax = 220 − age (Garber et al. 2011). Throughout the 25‐min exercise, the rate of RPE was recorded every 5 min.

### 2.5 Outcome Measures

#### 2.5.1 Pressure Pain Threshold

The participants were instructed to sit comfortably in an armrest chair and place their right palm up on the table. A handheld pressure algometer (FPX 25, Wagner Instruments Inc., Greenwich, CT) was applied to deliver pressure pain with a surface area of 1 cm^2^. A gradually increasing pressure was exerted vertically downward at a rate of approximately 1 N/s to the following two body sites on the dominant limbs: the midpoint of the line between the midpoint of the wrist and elbow (remote muscle site, described as PPT_forearm_) and the distal part of quadriceps muscle 10 cm superior the patellar bone (local muscle site, described as PPT_leg_). PPT was evaluated by escalating the body surface contact pressure until the participants' oral report of perceived pain and then recording it in Newtons. The measurements for each site were repeated three times with an interval of 15 s. The final PPT value was recorded as the average of the three values. To avoid numeric value bias, all assessments were conducted by one experimenter, who has extensively practiced applying this device before the measurement, and the display screen on the device was consciously blocked during the process of measurement.

#### 2.5.2 CPT and Tolerance

The participants were requested to immerse their right hands into the cold circulation water tank (TMS8032‐R15, Feidi Inc., Guangzhou, China). The temperature was maintained at an exact point (10°C) with a temperature stability of ± 0.1°C. The temperature settings were based on previous studies (Barati et al. 2017; Pourshoghi et al. 2016) and the results of the preliminary experiment of this study. The participants were required to keep their five fingers open and the immersion depth to the wrist stripes of the hand into the continuously circulated water, prohibiting touching the bottom and sides of the water tank, to ensure they felt the temperature evenly around their hands. Participants were reminded to leave their hands in the cold water as long as possible. The time was recorded in seconds, from the participants immersing their hands in cold water until they first felt pain (recorded as CPT) and could no longer withstand it (recorded as cold pain tolerance). Considering the safety issue and confounding effect, participants were not informed of the maximum length of time they could hold their hands in cold water until they reached the 4‐min limit and were asked to stop (Bowler et al. 2017).

#### 2.5.3 fNIRS Measurement During Cold Pressor Test

The hemodynamic changes (HbO_2_ signal) in the prefrontal cortex and bilateral motor cortex were monitored via a portable 35‐channel fNIRS device (NirSmart II, Danyang Huichuang Medical Equipment Co. Ltd., Danyang, China) with continuous waves (730 and 850 nm) during the process of the cold pressor test. Two circulation water tanks (TMS8032‐R15, Feidi Inc., Guangzhou, China) were used in the cold pressor test (one tank maintained a temperature of 10 ± 0.1°C and another maintained a temperature of 23 ± 0.1°C). The participants were asked to adjust the seat height to ensure eye level in line with the computer screen and to maintain a distance of approximately 65 cm away from it (Gajsar et al. 2020). For each trial of this test, participants were required to immerse their dominant hands into the continuously circulated cold water (10 ± 0.1°C) for 20 s after experiencing a baseline of 60 s for adaptation, followed by recovery with tepid water (23 ± 0.1°C) for 30 s. A total of 12 serial trials of the cold pressor test were performed in tepid water and cold water conditions. The block diagram of this protocol is shown in Figure 1B.

### 2.6 fNIRS Data Processing and Analysis

**FIGURE 3:**
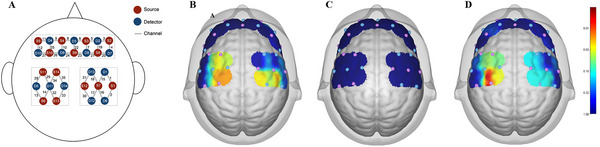
Schematic illustration of activated brain areas. (A) fNIRS optode layout (Sources: red; Detectors: blue). (B) Active HD‐tDCS group HbO2 changes. (C) Sham HD‐tDCS group HbO2 changes. (D) Between‐group difference comparison. Color scale indicates *p* values (*p* < 0.05 significant).

NirSpark software (Huichuang, China) was used to analyze the data on the changes in HbO_2_ obtained from fNIRS in accordance with the modified Beer‐Lambert law (Baker et al. 2014). The sampling rate was set to 11 Hz. The probe holders, including 14 emission sources and 14 detectors, were covered by alternating arrangements on the prefrontal cortex and bilateral motor cortex, resulting in 35 channels (Figure 3A). The inter‐probe distance was approximately 3 cm. On the basis of the 10–20 international standard system (Jurcak et al. 2007), the center of the probe matrix was placed over FPz, corresponding to D3 channel. Likewise, D11 and S7 channels were placed over C3 and C4, respectively, to maintain bilateral symmetry. The acquired coordinates were transformed into Montreal Neurological Institute (MNI) coordinates and further projected to the MNI standard brain template by using the MNI‐to‐Brodmann Atlas Tool in the NirSpark package, and then the Brodmann Atlas label of brain locations corresponding to each channel was obtained (Tsuzuki and Dan 2014).

Data preprocessing and analysis were divided into three parts: preprocessing, individual timeline analysis, and group‐level analysis. First, data preprocessing involved removing the motion artifacts and time interval unlinked with the experimental data in fNIRS raw data, including converting light intensity signal into optical density signal; adopting the spline interpolation method to correct the time window with motion artifacts; applying a band‐pass filter in the range of 0.01–0.2 Hz to remove physiological noise caused by respiration, heartbeat, and Mayer waves; and obtaining the changes in HbO_2_ by using the modified Beer‐Lambert law. In the individual timeline analysis, the hemodynamic responses of individuals during the cold pressor test were calculated. The inter‐trial changes in the difference between “2–12 s” during cold pain stimulation (as the HbO_2_ signal of peak) and “45–50 s” during tepid water washing‐out (as baseline) were also calculated. Then, 12 block paradigms were superposed and averaged. Finally, further statistical analysis was conducted in group‐level analyses.

### 2.7 Statistical Analysis

IBM SPSS Statistics 22.0 (SPSS Inc., IL, USA) was used for statistical analysis of all data, and GraphPad Prism 8 was used for depicting graphs. Descriptive statistics were presented as mean (standard deviations) for continuous variables, and categorical variables were presented as frequencies, percentages, or quartiles. In terms of data analysis, the Shapiro–Wilk normality test and Levene's homogeneity of variance were performed for continuous variables. Depending on the normality of data distribution, the Mann–Whitney *U* test or Student's *t*‐test was used to compare between‐group differences. Wilcoxon signed rank test or paired sample *t*‐tests were used to compare intra‐group differences. Fisher's exact test was used for categorical variables. For the physiological indices during exercise, the two‐way repeated measure analysis of variance (ANOVA) was used for intra‐group comparison. In addition, the relationship between primary and secondary outcomes (after intervention − before intervention) and the changes of HbO_2_ in the active and sham groups were assessed using Pearson's or Spearman's correlation analysis. The significance level was set as *α* = 0.05, two‐tailed.

## 3 Results

### 3.1 Baseline Characteristics of Participants

A total of 43 out of 46 eligible participants were recruited in this study. Four subjects were excluded due to poor data quality of the fNIRS signal. Hence, a total of 39 participants (21 males and 18 females) were eventually included in this analysis (Table 1). Baseline variables between the two groups (active HD‐tDCS and sham HD‐tDCS) were compared. No significant differences were found in demographic characteristics (age, gender, years of education, and BMI), psychological conditions (BDI‐PCS, SAI, TAI, FPQ, and PACES), and physical activity levels (total physical activity volume and sedentary time; all *p* > 0.05).

**TABLE 1:** Baseline information of the two groups.

	Total (*n* = 39)	Active HD‐tDCS (*n* = 19)	Sham HD‐tDCS (*n* = 20)	*p* value
**Age (years)**	**22.64 (2.67)**	**22.42 (1.68)**	**22.85 (3.39)**	0.623
Gender (men), *n* (%)	21(53.85)	11(57.89)	10 (50)	0.621
Education (years)	15.00 (0.00)	15.00 (0.00)	15.00 (1.50)	0.834
BMI (kg/m^2^)	21.60 (3.77)	20.78 (2.52)	21.96 (5.67)	0.509
BDI‐II	4.00 (7.00)	5.00 (7.00)	4.00 (8.50)	0.932
PCS	5.00 (16.00)	2.00 (17.00)	8.50 (15.75)	0.411
SAI^a^	36.44 (8.17)	33.84 (7.14)	38.90 (8.48)	0.052
TAI^a^	37.97 (9.75)	37.11 (9.08)	38.80 (10.51)	0.594
FPQ	75.00 (23.00)	76.00 (25.00)	74.00 (26.75)	0.261
Total PA (MET min/week)	3539.00 (4729.00)	3480.00 (3958.00)	3742.00 (5419.00)	0.779
Sedentary time (min)^a^	2219.49 (1048.13)	2034.21 (1001.12)	2395.50 (1086.62)	0.288

*Note*: Other continuous variables not normally distributed are expressed as medians (interquartile range); Categorical variables are presented as frequency (%);

Abbreviations: BDI‐II, Beck depression inventory‐II; BMI, body mass index; FPQ, fear of pain questionnaire; HD‐tDCS, high‐definition transcranial direct current stimulation; ME, metabolic equivalent; PA, physical activity; PCS, pain catastrophizing scale; STAI‐S, state trait anxiety inventory‐state; STAI‐T, state trait anxiety inventory‐trait.

^a^ Represents data normally distributed with equal variance, describes as mean (standard deviation).

### 3.2 Pain Sensitivity

**FIGURE 4:**
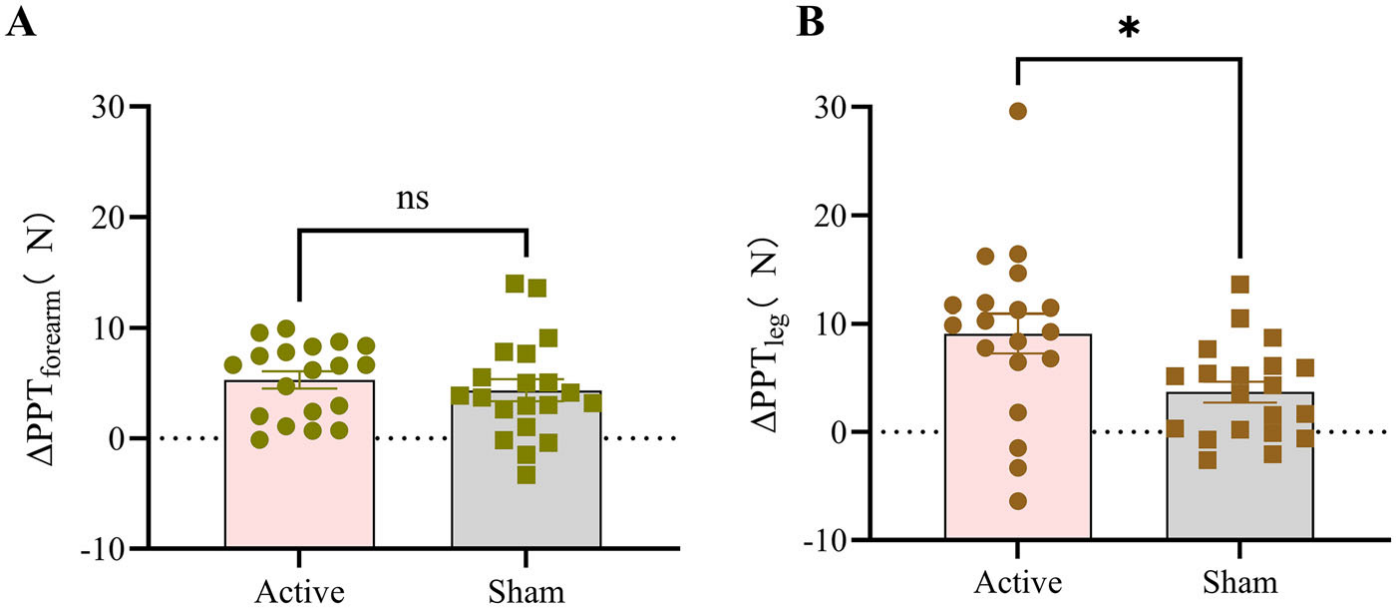
The inter‐group comparison of the difference values of PPT. (A) Between‐group differences in PPT_forearm_; (B) Between‐group differences in PPT_leg_; ΔPPT: Pre‐to‐post‐intervention change in PPT; Active: Active HD‐tDCS group; Sham: Sham HD‐tDCS group; * denotes statistical significance (*p* < 0.05); Data are expressed as mean ± SEM.

To account for baseline heterogeneity, we implemented analysis of covariance (ANCOVA) models with pre‐to‐post exercise change scores (Δ values) as dependent variables, including PPT_forearm/leg_, CPT, and cold pain tolerance. The models specified group allocation (Active HD‐tDCS vs. Sham HD‐tDCS) as a fixed factor, while incorporating baseline sensory measurements (PPT_forearm/leg_, CPT, and tolerance baseline values) as covariates. As illustrated in Figure 4, comparative analysis revealed a significant between‐group difference in PPT_leg_ Δ values for the Active HD‐tDCS group versus Sham controls (*F* = 6.731, *p* = 0.014). No other observed parameters reached statistical significance (all *p* > 0.05). The complete data can be found in Table 2.

**TABLE 2:** Changes of pain indices across different stimulation conditions.

Group	Number of examples	Change difference	Mean difference (95%CI)	F	*p* ^a^	*p* ^b^
**PPT_forearm_**						
Active HD‐tDCS	19	5.30 ± 3.34	1.024 (−1.488, 3.535)	0.683	0.414	< 0.001
Sham HD‐tDCS	20	4.35 ± 4.48				0.001
**PPT_leg_**						
Active HD‐tDCS	19	9.08 ± 8.01	5.404 (1.179, 9.628)	6.731	0.014	< 0.001
Sham HD‐tDCS	20	3.69 ± 4.36				0.004
**CPT (s)**						
Active HD‐tDCS	19	1.80 ± 2.42	0.186 (−1.365, 1.737)	0.059	0.809	0.082
Sham HD‐tDCS	20	1.57 ± 2.32				0.147
**Cold pain tolerance (s)**						
Active HD‐tDCS	19	1.30 ± 9.70	−1.022 (−6.463, 4.419)	0.145	0.705	0.732
Sham HD‐tDCS	20	2.58 ± 6.84				0.204

*Note*: Data are expressed as mean ± SD; PPT_forearm_ and PPT_leg_ means the midpoint of the line between the midpoint of the wrist and elbow, and the distal part of quadriceps muscle 10 cm above the patellar bone, respectively. *p* < 0.05 indicates a statistically significant difference.

Abbreviation: HD‐tDCS, high‐definition transcranial direct current stimulation.

^a^ Statistical analyses were performed using ANCOVA with baseline PPT values as the covariate.

^b^ Represents pre‐to‐post exercise, normally distributed data was analyzed using the paired *t*‐test, while non‐normally distributed data was assessed with the Wilcoxon signed‐rank test.

### 3.3 fNIRS Results

For the active HD‐tDCS group, with‐group analysis showed significant differences in the following channels: CH16 (*t* = −3.535, *p* = 0.026), CH30 (*t* = −3.243, *p* = 0.037), CH32 (*t* = −3.703, *p* = 0.026), CH33 (*t* = −4.034, *p* = 0.025), and CH34 (*t* = −3.147, *p* = 0.037), corresponding to the left supplementary motor cortex, pre‐motor cortex, and the right primary somatosensory cortex (S1) (Figure 3B). No significant within‐group difference was found in the sham HD‐tDCS group (Figure 3C).

The comparison of results between groups (as shown in Figure 3D) revealed that compared with the sham HD‐tDCS group, the active HD‐tDCS group showed significant differences in CH32 (*t* = −3.047, *p* = 0.004), CH33 (*t* = −2.216, *p* = 0.033), and CH34 (*t* = −2.069, *p* = 0.046). That is, compared with aerobic exercise, adding HD‐tDCS stimulation during exercise significantly activated the left premotor and supplementary motor cortices on the left side.

**FIGURE 5:**
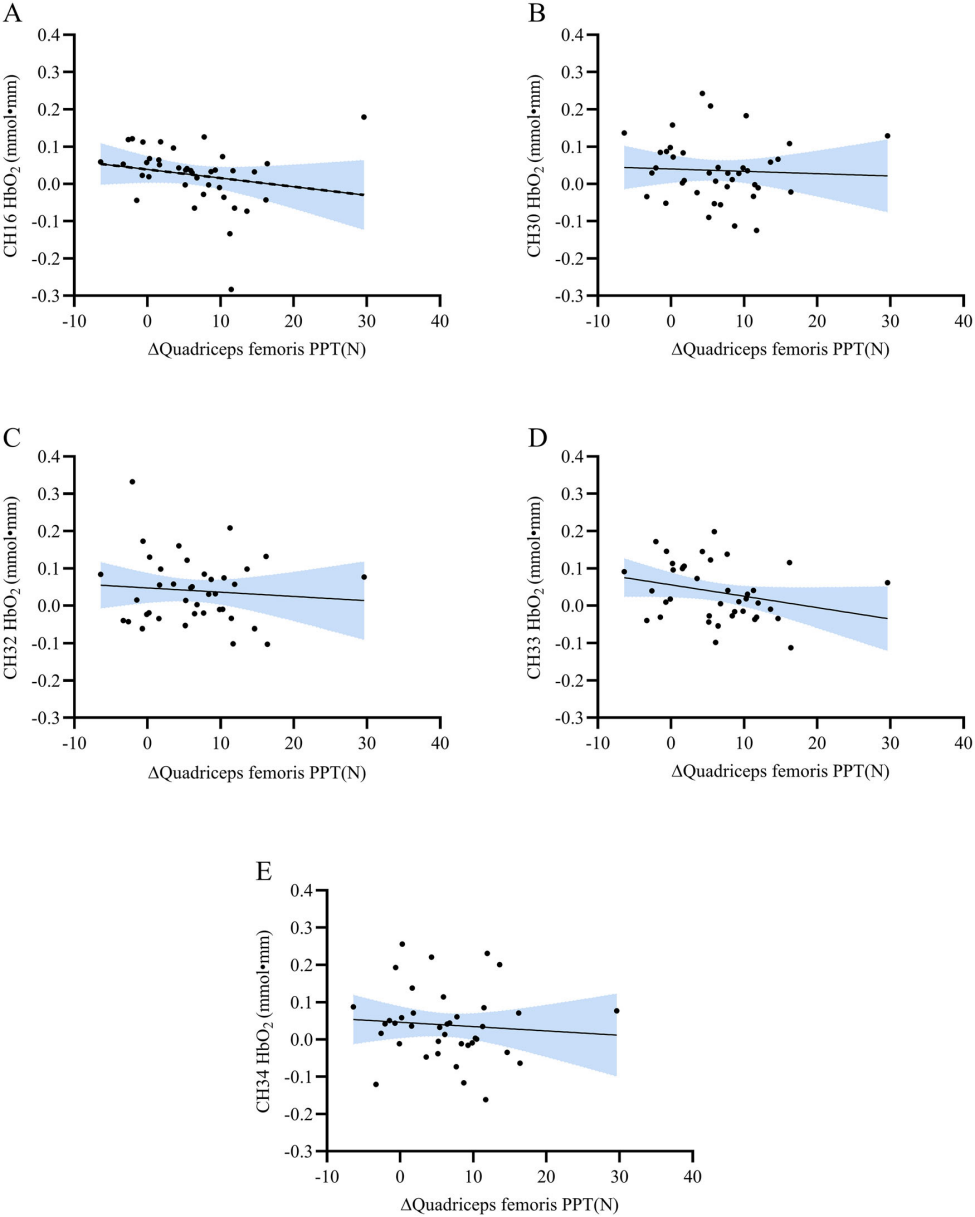
Correlation between the difference in PPT and changes in HbO_2_. (A) Correlation analysis between CH16 and quadriceps PPT; (B) Correlation analysis between CH30 and quadriceps PPT; (C) Correlation analysis between CH32 and quadriceps PPT; (D) Correlation analysis between CH33 and quadriceps PPT; (E) Correlation analysis between CH34 and quadriceps PPT.

Spearman correlation analysis between cortical activation in motor cortex channels (CH30, CH32, CH33, and CH34) and S1 (CH16) with PPT_leg_ revealed a negative association at CH16 (*r* = −0.405, raw *p* = 0.011). After Benjamini–Hochberg correction for multiple comparisons across all five channels, this association showed a trend toward significance (adjusted *p* = 0.055) (Figure 5). Motor cortex channels demonstrated no notable associations (adjusted *p* > 0.2).

### 3.4 Blinding and Stimulation Tolerance

Immediately after HD‐tDCS intervention, the efficiency of blinding and adverse effects were assessed via a questionnaire (asking participants about the type of stimulation they received and to choose which of the following types of discomfort occurred and the degree of symptoms during the process of stimulation: headache, neck pain, scalp tingling, and scalp burning, among others). Among the 39 participants, 22 (56.41%) correctly guessed the stimulation types, and Fisher's exact test results showed no significant difference in the distribution between the right and wrong guesses (*p* = 0.751). As shown in Table 3, scalp tingling was the most reported (the active HD‐tDCS group accounted for 42.11% and the sham HD‐tDCS group accounted for 35%). Fisher's exact test showed no significant difference between the two groups (*p* = 0.748).

**TABLE 3:** Adverse effects after active and sham HD‐tDCS stimulation.

	Total (*n* = 39)	Active HD‐tDCS (*n* = 19)	Sham HD‐tDCS (*n* = 20)	*p* value^a^
Headache	1(2.56)	0 (0)	1 (5)	1.000
Neck pain	2 (5.13)	1 (5.26)	1 (5)	1.000
Scalp pain	12 (30.77)	6 (31.58)	6 (30)	1.000
Tingling	6 (15.38)	4 (21.05)	2 (10)	0.407
Itching	15 (38.46)	8 (42.11)	7 (35)	0.748
Skin redness	3 (7.69)	2 (10.53)	1 (5)	0.605
Sleepiness	2 (5.13)	1 (5.26)	1 (5)	1.000
Trouble concentrating	4 (10.26)	2 (10.53)	2 (10.53)	1.000
Acute mood changes	0 (0)	0 (0)	0 (0)	1.000
Others (specific)	0 (0)	0 (0)	0 (0)	1.000

*Note*: Data are expressed as frequency (%).

^a^ Two‐sided Fisher's exact test.

### 3.5 Data Collection During Exercise

**FIGURE 6:**
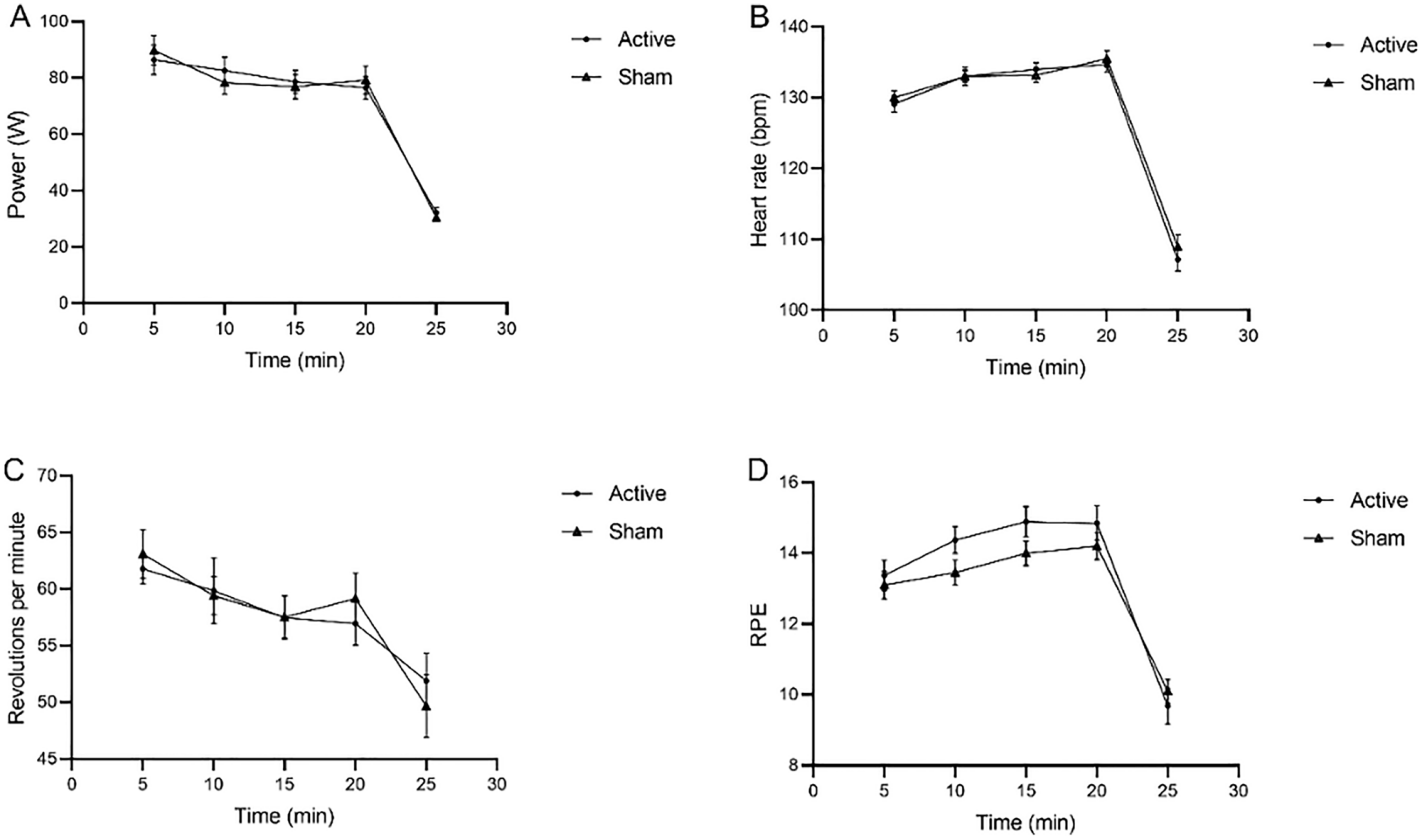
The trends of physiological indicators during exercise with respect to time changes. Active: Active HD‐tDCS group; Sham: Sham HD‐tDCS group.

A two‐way repeated‐measures ANOVA was performed to analyze the temporal trends of power, HR, revolutions per minute (RPM), and RPE recorded every 5 min during exercise (as shown in Figure 6). First, boxplot analysis confirmed no outliers in the data. Second, Shapiro–Wilk tests indicated normal distribution for all variables across groups (*p* > 0.05). Finally, Mauchly's test revealed violations of sphericity for all four outcomes (*p* < 0.05); thus, between‐subjects effects were examined. Results demonstrated no statistically significant differences between groups for any outcome: power (*F* = 0.006, *p* = 0.939), HR (*F* = 0.216, *p* = 0.645), RPM (*F* = 0.005, *p* = 0.944), or RPE (*F* = 1.240, *p* = 0.273).

## 4 Discussion

In the present study, the main findings confirmed the facilitatory effect of tDCS on EIH induced by moderate‐intensity aerobic exercise among healthy individuals. Specifically, a significant increase in PPT at the local site was observed in the active tDCS group compared with the sham group. The neuroimaging results revealed by fNIRS showed significant differences in neuronal activity changes between the two groups in brain areas, including the left supplementary motor cortex and pre‐motor cortex. Concurrently, the changes in pain sensitivity indicators were significantly negatively correlated with the alterations in brain activation.

In terms of EIH, both the active tDCS group and sham group exhibited an improvement in PPT at local and remote sites following medium‐intensity aerobic exercise, consistent with previous EIH studies (Vaegter et al. 2018, 2014). However, the active HD‐tDCS group demonstrated a significantly greater increase in local PPT_leg_ compared to sham stimulation, which suggested that active HD‐tDCS strengthened EIH (Vaegter et al. 2014; Koltyn et al. 2014; Naugle et al. 2012). This enhancement in local pain modulation holds direct clinical relevance: by amplifying exercise‐derived analgesia, HD‐tDCS could improve patients' tolerance to therapeutic movements (for e.g., postinjury rehabilitation or chronic pain exercises), thereby increasing adherence to rehabilitation protocols and reducing pain‐related activity avoidance—a critical barrier in functional recovery. The absence of between‐group differences in PPT at remote sites suggests that HD‐tDCS selectively enhances local EIH rather than systemic pain modulation. This distinction aligns with evidence that local EIH involves sensory‐motor cortical plasticity, whereas remote hypoalgesia is predominantly mediated by endogenous opioid release (Micalos and Arendt‐Nielsen 2016; Gurdiel‐Álvarez et al. 2023). However, it should be noted that not all studies have observed synergistic effects of tDCS and exercise on pain modulation. For instance, Lewis et al. (2024) reported no significant differences in pain pressure thresholds between active and sham tDCS when combined with an isometric grip strength task in healthy adults. One possible explanation is the difference in exercise modality. Isometric grip tasks may elicit weaker or more localized hypoalgesic effects compared to aerobic exercise, and may reach a ceiling effect, leaving little room for tDCS to enhance pain modulation. In contrast, moderate‐intensity aerobic exercise, as employed in the present study, is more likely to engage broader descending inhibitory systems, providing a more favorable physiological context for HD‐tDCS to exert a synergistic effect. These discrepancies underscore the importance of considering exercise type when designing tDCS‐exercise interventions for analgesia. Similarly, our study found no significant HD‐tDCS effects on CPT. The discrepancy may relate to the types of nerve fibers mediating EIH. Cold pain is predominantly transmitted via Aδ fibers, whereas pressure pain is mediated by unmyelinated C fibers. In addition, differences in experimental paradigms may contribute to the observed results. Gurdiel‐Álvarez et al. (2023). uggest that the tonic cold pain stimulation paradigm—assessing CPT by immersing the hand in cold water and recording the time until pain onset—differs from phasic cold pain stimuli used in quantitative sensory testing. Tonic stimuli evoke greater unpleasantness and involve more emotional processing compared to phasic stimuli, which may have confounded the outcomes. Furthermore, postexercise changes in skin temperature could play a role. Studies indicate that exercise‐induced alterations in skin impedance and temperature complicate precise measurement of pain threshold changes using electrical or cold stimuli (Sato et al. 2021). Thus, PPT is a more reliable indicator of exercise‐related pain sensitivity than CPT.

Our study discovered that active HD‐tDCS augmented the analgesic effect following aerobic exercise. Furthermore, the possible underlying mechanisms were explored using fNIRS neuroimaging. Significant activations in the left SMC and pre‐MC regions were seen in the active HD‐tDCS group, in relation to the sham group. Besides the two brain areas, the right S1 was activated in within‐group analysis. Interestingly, a “functional targeting” phenomenon was observed, which meant that the activation of the neural network on the contralateral side (non‐stimulation side) was diminished (Bikson et al. 2013). In addition, tDCS stimulation may be related to adaptive changes in pain avoidance behavior, which is normally associated with sensory‐motor cortex activation (Abram et al. 2022). At the same time, there were also significant associations between the pain sensitivity presentations and the brain plasticity changes, indicating those with lowered pain sensitivity were more likely to have greater analgesic effect upon tDCS combined with aerobic exercise. It is suggested that pre‐exercise tDCS stimulation plays a “top‐down” role in priming the pain inhibitory system, while the aerobic exercise followed produces EIH from a “bottom‐up” approach (Cardenas‐Rojas et al. 2020; Duarte et al. 2018). HD‐tDCS may be a promising adjunct to facilitate EIH for pain management. We observed a nominally negative association between PPT_leg_ and activation in the right S1 cortex (CH16) during pain induction. Thding is consistent with previous similar studies (Sorkpor et al. 2023). This might indicate that the weakened ability of perceiving and discriminating the perception of harmful signals could be a potential reason for the enhancement of the phenomenon of pain attenuation. This is consistent with the role of S1 in encoding the intensity and spatial location of pain. Although the CH16 correlation did not reach strict statistical significance under FDR control, the moderate effect size (*r* = −0.405) suggests that it may have clinical relevance. However, given that the statistical support is at the verge, caution should be exercised when interpreting the results, and verification in a larger cohort is needed.

There are several strengths in the study. This study provides the first ​neurophysiological evidence combining HD‐tDCS and fNIRS to delineate cortical mechanisms underlying EIH enhancement. Second, the HD‐tDCS was employed for stimulation in the present study, which has a superior effect over traditional tDCS in terms of targeting accuracy and treatment safety (Villamar et al. 2013; DaSilva et al. 2015). In addition, the left M1 region was selected as the target region for tDCS stimulation, which is widely recognized based on the most updated evidence on pain modulation using noninvasive brain stimulation methods (Chang et al. 2018, 2015).

This study has several limitations as well. First, while our findings in healthy individuals provide foundational insights into HD‐tDCS mechanisms, caution is warranted when extrapolating these results to chronic pain populations, where EIH is often impaired (Rice et al. 2019). However, studying healthy participants allows precise isolation of neuromodulatory effects without confounding factors such as central sensitization or comorbidities, establishing a critical benchmark for future clinical trials. Second, while participants were blinded to group allocation, the intervention administrators were unavoidably unblinded due to the technical requirements of the equipment. Importantly, outcome assessors and data analysts remained fully blinded throughout the study. To minimize bias, PPT assessments were standardized and administered by a single trained experimenter. Future research can adopt fully automatic pressure gauges to further eliminate operator‐related variability. At the same time, the parameters of HD‐tDCS (such as intensity, duration, or cortical targets) should be prioritized for optimization. Finally, longitudinal studies in clinical populations are crucial for verifying the translational potential of this integrated approach and evaluating its persistence beyond the acute experimental environment.

## 5 Conclusion

Anodal HD‐tDCS over the left M1 enhanced EIH among healthy individuals. The augmented effect was associated with increased activation in cortical areas concerning sensory‐motor processing. Further study is warranted to explore the effect of tDCS on EIH in those with chronic pain.

## Author Contributions

**Ruihan Wan**: conceptualization, formal analysis, data curation, writing – original draft, methodology. **Haozhi Zhao**: methodology, writing – original draft. **Xue Jiang**: writing – original draft, writing – review and editing. **Beibei Feng**: writing–original draft, writing – review and editing. **Yafei Wang**: validation, visualization. **Chen Gong**: writing – review and editing. **Yangyang Lin**: writing – review and editing. **Wangwang Yan**: writing – review and editing. **Yixuan Ku**: conceptualization, supervision, writing – review and editing. **Yuling Wang**: writing – review and editing, supervision, conceptualization.

## Ethics Statement

The study was performed in accordance with the Declaration of Helsinki. It was approved by the Ethics Committee of the Sixth Affiliated Hospital of Sun Yat‐sen University, Guangzhou, China (2021ZSLYEC‐350).

## Consent

Informed consent was obtained from all individual participants included in the study.

## Conflicts of Interest

The authors declare no conflicts of interest.

## Peer Review

The peer review history for this article is available at https://publons.com/publon/10.1002/brb3.70595.

## Supporting information




**Supporting Material**: brb370595‐sup‐0001‐SuppMat.docx

## Data Availability

Data will be made available on request.
