# Supplementary figures and images for: High‐Definition Transcranial Direct Current Stimulation Enhances Exercise‐Induced Hypoalgesia in Healthy Individuals: An fNIRS Study
Source: Brain Behav. 2025 Jun 10;15(6):e70595. doi: 10.1002/brb3.70595 (PMC12152266; doi:10.1002/brb3.70595)

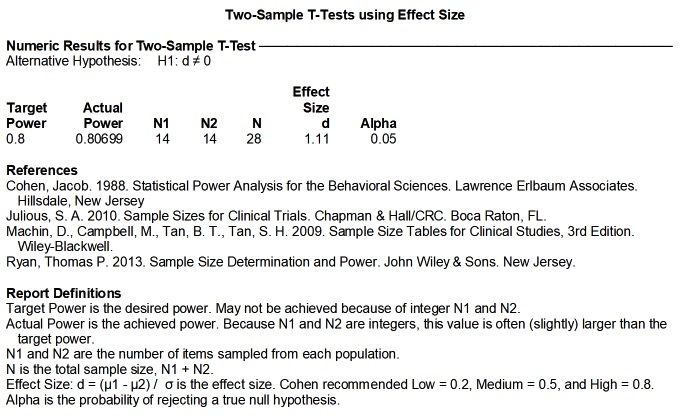
S1. Sample size calculation

Supplement: Supplementary file 1 — Supporting Material: brb370595‐sup‐0001‐SuppMat.docx [file BRB3-15-e70595-s001.docx]
